# The European roadmap towards fusion electricity

**DOI:** 10.1098/rsta.2017.0432

**Published:** 2019-02-04

**Authors:** A. J. H. Donné

**Affiliations:** EUROfusion, Programme Management Unit, Boltzmannstrasse 2, 85748 Garching, Garmany

**Keywords:** nuclear fusion, ITER, DEMO, tokamak

## Abstract

The European roadmap to the realization of fusion electricity breaks the quest into eight missions. For each mission, it reviews the current status of research, identifies open issues, and proposes a research and development programme. ITER is the key facility on the roadmap as it is expected to achieve most of the important milestones on the path to fusion power. The Fusion Roadmap is tightly connected to the ITER schedule and the vast majority of resources in fusion research are presently dedicated to ITER and its accompanying experiments. Parallel to the ITER exploitation in the 2030s, the construction of the demonstration power plant DEMO needs to be prepared. DEMO will for the first time supply fusion electricity to the grid and it will have a self-sufficient fuel cycle. The design, construction and operation of DEMO require full involvement of industry to ensure that, after a successful DEMO operation, industry can take responsibility for commercial fusion power. The European fusion roadmap provides a coherent path towards the fusion power plant, and it proposes in an integrated way to find solutions for all challenges that still need to be addressed.

This article is part of a discussion meeting issue ‘Fusion energy using tokamaks: can development be accelerated?’

## 1. Introduction

There is a general understanding that mankind needs to strongly reduce its reliance on fossil fuels for energy generation. Partly this necessity arises from the limited available fossil fuel resources, but more importantly from the CO_2_ emissions which are the main driver behind global warming and climate change [1]. Many governments are at present strongly investing in renewable energy sources such as wind and solar, but due to the intermittent nature of these sources in combination with the lack of efficient storage options, the share these renewable sources can have in the energy mix is limited [2–5].

Thanks to enormous subsidies for solar and wind energy in Germany, amounting to on average 25 billion € per year in recent years, wind and solar contribute nowadays to about 40% of the German electricity mix. However, surprisingly, this has had a negligible effect on CO_2_ emissions due to electricity generation (www.cleanenergywire.org/news/german-co2-emissions-rise-2015-despite-renewables-surge). The reason for this is that large-scale back-up plants are needed for the majority of the days that there is not enough wind or sun. Since nuclear plants are also being phased out in Germany after the Fukushima event in 2011, the reliance on fossil fuels as a backup source has become even stronger. Developing proper storage options for intermittent sources that can cope with both the nocturnal as well as the seasonal variations will certainly improve the situation. Nevertheless, even with the best possible storage means and making use of a European super grid the relative contribution of renewables to the electricity mix is estimated to be still limited to a maximum of about 60% [5]. This is in line with the IPCC Special Report on Renewable Energy Sources and Climate Change Mitigation that says: ‘more than half of the scenarios show a contribution from renewable energies in excess of a 17% share of primary energy supply in 2030, rising to more than 27% in 2050. The scenarios with the highest renewable energy share reach approximately 43% in 2030 and 77% in 2050’ [6].

To further reduce the usage of fossil fuels for generating electricity one needs more options than are presently considered in the renewable energy mix. There is, therefore, room, and possibly need, for nuclear energy as part of the energy mix: fission or fusion. Nuclear fusion has the advantage that the fuel constituents (deuterium and lithium) are available in inexhaustible quantities, that there are no long-lived waste products and that the process is inherently safe. Nuclear fusion is the energy source of the sun, and it works. However, it has the disadvantage that it is very difficult to create an efficient fusion reactor on Earth. According to the European Fusion Roadmap, nuclear fusion will not be realized by 2050, but it has the potential to significantly contribute to the energy mix in the long term and especially to provide electricity (or heat) in densely populated countries and highly industrialized areas, where land scarcity hampers the utilization of intermittent sources.

Given the very positive prospects of nuclear fusion, in 2012 Europe drafted the European Fusion Roadmap to the realization of fusion energy [7]. The Fusion Roadmap describes in detail the research that needs to be done to lay the foundation for a Fusion Power Plant. Based on detailed assessments and reviews, an overview has been made of the status of the field and the open challenges, and a resource-loaded research plan has been drafted to address these. The Fusion Roadmap is broken up into eight missions/challenges which will be briefly described in this paper.

## 2. The European roadmap to fusion electricity

The fusion roadmap forms a credible basis for the European fusion programme. It provides a clear and structured way forward to a demonstration of commercial electricity production from magnetically confined fusion on a realistic timescale. The main facilities on the fusion roadmap are (1) ITER which should demonstrate that fusion is feasible, (2) DEMO which will have a self-sufficient fuel cycle and produce for the first time electricity from fusion and (3) IFMIF-DONES which is a 14 MeV neutron source needed to test and validate materials for DEMO and the fusion power plants.

ITER is aimed at reaching a ratio of thermal fusion power over input power *Q* = *P*_fus_/*P*_in_ of 10 with a deuterium-tritium mixture. This implies that conditions are reached in ITER in which the alpha particles have enough energy to dominantly heat the plasma. ITER will not generate electricity, but it will test the technologies to produce tritium from lithium in special Test Blanket Modules.

DEMO is the demonstration reactor that will produce for the first time electricity from the fusion process and operate with a closed tritium fuel-cycle (i.e. DEMO must produce its own fuel) [8]. The European DEMO design is at present a conservative extrapolation from ITER, using as far as possible technologies that have already been tested, which will ease the nuclear licensing process. In parallel, ways to improve the DEMO design using new physics and technology findings are explored. The target electrical output of DEMO will be in the range of 300–500 MW_E_.

The International Fusion Materials Irradiation Facility (IFMIF) is an accelerator-based neutron source [9] required to test and validate materials for DEMO and Fusion Power Plants under a fusion relevant neutron load and spectrum. The materials test facility should become operational in the second half of the 2020s such that the new materials are validated before DEMO is due to be built. Therefore, a lighter version, IFMIF-DONES (DEMO Oriented Neutron Source) [10], is presently being proposed by Europe as it can be constructed and operational in a shorter time.

Although ITER, DEMO and IFMIF-DONES are the main devices on the European Fusion Roadmap, many contemporary facilities including tokamaks (JET, ASDEX Upgrade, TCV, Mast-Upgrade, WEST), linear devices (Magnum-PSI, Pilot-PSI, Jule-PSI, Judith) and a stellarator (W7-X) are needed to address the challenges that form the eight missions, which are:

(1) Demonstrate plasma regimes of operation that increase the success margin of ITER and satisfy the requirements of DEMO;
(2) Demonstrate heat-exhaust systems capable of withstanding the large power loads in DEMO;
(3) Develop and validate neutron-resistant materials that can withstand the large 14 MeV neutron fluence without strongly degrading their physical properties;
(4) Ensure tritium self-sufficiency through technological solutions for the breeding blanket;
(5) Implementation of the intrinsic safety features of fusion into the design of DEMO following experiences gained with ITER;
(6) Produce an integrated DEMO design supported by targeted R&D activities;
(7) Ensure the economic potential of fusion by reducing the DEMO capital costs and developing long-term technologies;
(8) Bring the stellarator line to maturity to be able to judge its feasibility as a long-term backup solution.

It is necessary to solve in parallel the challenges in all missions to develop a fusion power plant producing electricity.

In the remainder of this paper, a brief overview is given of the challenges in the eight missions. To restrict the length of the paper, it is not possible to go into all aspects of each mission.

## 3. Developing plasma regimes of operation for ITER and DEMO

Achieving power from fusion requires the confinement of a high-performance plasma at high enough density and temperature for a long enough time. However, at the same time, any instabilities and transport processes that might arise in the plasma must be controlled. The power loads on the plasma facing components (which must be very thin in DEMO as the neutrons need to lose their energy in the breeding blanket) and in the divertor must be acceptable. For DEMO this implies typical wall loads less than 1 MW m^−2^ on the wall and less than 10 MW m^−2^ on the divertor. To make these possible, highly radiative plasmas must be engineered where most of the radiation is uniformly emitted from the plasma edge.

Carbon has been for a long time the standard material for the plasma facing component as it is rather forgiving when the plasma touches the wall. Unfortunately, carbon reacts easily with hydrogenic isotopes (including tritium) to form carbohydrates. Additionally, carbon easily forms dust with the result that much of the tritium would lay on the bottom of the tokamak. Therefore, in Europe, a clear decision was made towards metallic plasma facing components. This was first pioneered on ASDEX Upgrade which was gradually equipped with a tungsten wall and later followed by JET, where most of the wall was changed to beryllium, while the divertor was changed to tungsten. As a result, the tritium retention dropped by a factor of 20, to a level low enough for ITER to be able to work for a long time without any opening to get the tritium out [11,12]. A drawback of the metallic wall, however, is the fact that it is less straightforward to make high-performance plasmas, as the heavy metals from the divertor have a tendency to migrate into the core plasma. Special operational procedures are needed to achieve high-performance operation in a metal wall tokamak. This involves ample central heating, seeding of the edge plasma with noble gasses, flushing impurities with benign Edge Localized Modes, etc. Detailed studies at JET [13] over a number of years were needed to bring the performance with the ITER-Like Wall back to the same level as with the former Carbon wall. The effect of choking of the plasma performance due to influx of heavy metal impurities is, in general, less severe in a large tokamak with a larger volume to surface ratio.

The operation at high performance, near the operational limits, comes at a price. Namely, disruptions in which the plasma comes to a sudden termination lead to strong forces on the vacuum vessel [14]. These become stronger for larger devices and therefore much effort needs to be devoted to avoiding, suppressing and/or mitigating disruptions by using such techniques as massive gas and shattered pellet injection. Much effort is devoted to predict well in time whether a disruption has a likelihood to occur during the plasma discharge, such that measures can still be taken to avoid the disruption.

## 4. Heat exhaust systems

In ITER, the steady-state heat loads on the target plates in the divertor will be in the order of 10 MW/m^2^ under detached plasma conditions. Transiently, during Edge Localized Modes, the heat loads can reach levels of a few GW m^−2^ during pulses of typically 0.5–1 ms. Plasma-facing components need to withstand these heat loads for a sufficiently long time to avoid too frequent shutdowns to replace them. DEMO will feature much longer pulses, while it has much thinner walls than ITER (which comes from the fact that the neutrons should not lose their energy in the wall, but in the breeding blanket behind it) and therefore it is necessary to develop highly radiative plasmas for DEMO in which most of the outgoing energy is uniformly radiated from the plasma edge.

Mission 2 of the European Roadmap is based on three pillars:

(1) Develop routine and robust control systems for plasma detachment in a conventional divertor geometry;
(2) Develop, improve and test new divertor materials and plasma facing components;
(3) Test alternative magnetic geometries to spread the plasma heat load over a larger surface of the divertor.

JET and the three medium-sized tokamaks ASDEX Upgrade, TCV and MAST-Upgrade are being used to test divertor detachment as well as alternative divertor geometries. The ultimate test of controlled divertor detachment with a conventional divertor will be in ITER. During divertor detachment, the electron temperatures in front of the divertor are reduced to values below approximately 5 eV, such that recycled neutral particles from the target plates undergo a number of charge-exchange collisions with plasma ions before they are ionized [15]. As a result, the plasma flow to the target plates becomes more diffusive and is strongly reduced.

The TCV tokamak exploits the snowflake divertor geometry [16] in which the heat load from the plasma is split over four instead of two footprints, thus lowering the maximum heat load on the divertor plates. To enhance its flexibility, the divertor of TCV is being equipped with two high-*T*_c_ super-conducting coils. In MAST-Upgrade the Super-X geometry will be tested [17]. In this concept, the outer divertor plates are located at the largest plasma radius that is possible inside the toroidal field coils to increase the plasma-wetted area. ASDEX Upgrade in its turn will be equipped with an upper divertor such that it can study plasmas in the so-called double-null configuration. All three devices have the possibility to study multiple geometries. Finding the optimum divertor geometry is one part of the challenge; the second part is to find a divertor geometry that is DEMO-compatible as it makes no sense to use a divertor in DEMO that uses magnetic coils that are placed very close to the plasma and that are therefore very vulnerable to the neutron bombardment coming from the plasma.

Liquid metal targets are among the new materials that are being investigated [18]. They have the advantage that the divertor is in principle self-healing in case material is evaporated. Drawbacks are the possibility of droplets that may enter the plasma and the fact that the desired flow pattern might be affected by the magnetohydrodynamic forces in the tokamak. It will also be necessary to develop a scheme in which the loop of the liquid flow is closed so that the material cannot accumulate in the plasma or the vessel.

EUROfusion is exploiting a range of devices to test plasma-facing components. These include the super-conducting Magnum-PSI linear plasma simulator [19], which can mimic heat loads exceeding 10 MW m^−2^ in steady-state conditions as well as pulsed heat loads up to 1 GW m^−2^ to mimic Edge Localized Modes. JUDITH [20] can test materials with an intense electron beam, while the superconducting WEST tokamak [21] will test an actively cooled divertor under similar power loads as in ITER.

## 5. Neutron tolerant materials

The neutrons generated by the fusion reactions interact with the material structures surrounding the plasma. Largely this is desirable as the neutrons need to produce the tritium in the breeding blankets and also to generate the heat driving the electricity-generating turbines. However, the neutrons will also displace atoms in any structure close to the plasma. In ITER, every atom in the plasma facing materials will undergo on average typically 1–2 displacements (expressed in dpa, displacements per atom). The materials in ITER are designed to cope with these loads. DEMO will have neutron loads that are in the order of approximately 20 dpa during the first phase when it is operated with a starter blanket, and about 50 dpa in a later phase. It is not so much the neutron flux, but the neutron fluence (due to the longer pulses) that leads higher dpa level.

Materials that can be used in the first phase of DEMO already exist. They have been tested and validated in Materials Test Reactors. For the full qualification, they need to be further tested and validated in a fusion relevant neutron spectrum. Materials for the later phase of DEMO still need to be developed (or at least improved with respect to the present materials).

In Europe, the fusion materials research strongly concentrates on a limited number of main materials: Eurofer, a reduced-activation ferritic martensitic steel alloy, for the structural components; tungsten for the plasma facing components; and CuCrZr for the components aimed at removing most of the power deposited in the plasma facing structures. An issue is that many of the materials suffer from embrittlement at low temperatures, while the mechanical strength deteriorates at too high a temperature. Therefore, much work is being devoted to widening the operational window of the materials. A relatively recent success is the widening of the operational window of Eurofer97-2 by applying specific non-standard heat treatments [22]. For cooling materials, an important issue is the loss of mechanical strength of CuCrZr above 300°C under irradiation. Much attention is devoted to reinforcement strategies to extend the use of materials from ITER to the much more demanding DEMO operating conditions [23]. Good progress has been reported with fibre-reinforced CuCrZr pipes, in which tungsten wires are braided and then embedded in the alloy by melt infiltration.

For the validation and qualification of materials to be used in a fusion reactor, irradiations campaigns in test reactors are being executed and need to be continued in the coming decade. The ultimate testing and validation should be done with a source featuring a fusion-relevant neutron spectrum. Such a source is the IFMIF [9] or the DONES [10]. Both neutron sources are based on a deuterium ion accelerator directed towards a liquid lithium target, where the Li(d,xn) nuclear reaction will yield a neutron spectrum that is reminiscent of that of a fusion reactor. The main difference between IFMIF and DONES is that IFMIF has two accelerators, whereas DONES has only one. The design of DONES is presently ongoing and construction should start early in the next decade. The preferred site in Europe is near Granada in Spain.

The neutron wall load is an important measure of the severity of the operational environment for the first-wall and blanket. Higher loads need more cooling which has an adverse effect on the Tritium Breeding Ratio. Additionally, there are strong limitations arising from available fusion nuclear-grade structural materials. Materials that can withstand loads above typically 20 dpa still have to be developed, tested and qualified. Typically, 30 years are needed from the early development of a material until the final qualification (necessary to obtain the nuclear license).

## 6. Tritium self-sufficiency

Tritium, one of the fuel constituents, does not occur readily in nature as it has a very short radio-active decay time of 12.32 years. It can be produced in the CANDU type of nuclear fission reactors, but the commercially available quantities are only in the order of several tens of kilogram [24]. Therefore, the tritium needed as fuel should be produced in the reactor itself. This can be done by surrounding the plasma with a blanket containing ^6^Li, such that the escaping neutrons produce the tritium via the ^6^Li(n,^3^H)^4^He reaction. The tritium and helium are separated from the lithium; the tritium is fed back into the plasma, whereas the helium is a valuable non-radioactive and non-toxic exhaust product. For a fusion reactor to be economically viable the tritium used as fuel should be completely replenished. In other words, the tritium breeding ratio should be at least 1. Ideally, it should be 1.05 or even a little higher to compensate for some tritium losses (e.g. due to sticking to the metal wall) and also to produce a start-up quantity for the next generation of fusion reactors.

Tritium production will be tested in a number of different test blanket modules in ITER to find out which of the concepts is the most viable. Europe is working on four different concepts for the DEMO blanket with different levels of design/technology readiness, based on water, helium and LiPb as coolants and a solid or LiPb as tritium breeder/neutron multiplier [25]. It is proposed that two of these concepts will be tested in ITER: the helium-cooled pebble bed (HCPB) and the water-cooled lithium loop (WCLL).

The thickness of the blanket needed for achieving a tritium breeding ratio larger than 1 and at the same time screen the superconducting magnetic field coils from the neutrons is independent of the tokamak size and the magnetic field. In case tritium breeding at the high-field side is needed, the distance between the inner leg of the toroidal field coil and the plasma (made up of thermal shield, vacuum vessel, breeder and neutron shielding, first wall and scrape-off-layer) cannot be much smaller than 1.7 m. The development of tritium breeding concepts is complicated and the first results of the various concepts will only become available when ITER reaches its high-performance phase. A fusion reactor that is claimed to come into operation before ITER reaches high performance will, therefore, miss this input and might not be able to breed its own tritium, but instead use tritium from external sources.

## 7. Safety and environment

DEMO and the future Fusion Power Plants are nuclear devices. This implies that safety is an issue in all sub-projects from the first day of conceptual design onwards. Despite the fact that a fusion reactor is inherently safe, everything possible should be done to protect the workers and the people living in the environment from any risk. To obtain a license to operate the device it must be demonstrated to the nuclear regulator that all aspects of the reactor are safe and that there are no hidden pitfalls [26]. Having the present negative public opinion in mind about the employment of nuclear fission plants it is important to convince society that the risks associated with the operation of a fusion plant (e.g. the handling of tritium, the handling of short-lived nuclear waste, etc.) are well under control. Safety should not only comprise safe operation of the fusion reactor but also safe remote maintenance procedures. Care should be taken that the volume contaminated by radioactive material (including tritium) is as small as possible. The extraction, handling and storage of in-vessel components (activated and T-contaminated) are complex operations requiring extensive tools and infrastructure. Such operations can hardly be qualified as ‘plug and play’.

## 8. Integrated DEMO design

The aim of the European Fusion Roadmap is to demonstrate fusion electricity to the grid early in the second half of the century. This implies that the Engineering Design Activity of DEMO should begin before ITER starts its high-performance operation phase. Therefore, a thorough analysis has been made of which elements of DEMO can be only finalised after demonstration in ITER, and which parts of the DEMO design can already be tackled at an earlier phase. Some elements of the DEMO design could allow for a range of ITER outcomes, to have somewhat more flexibility.

The present DEMO pre-conceptual design activity comprises:

(1) A strong philosophy of systems engineering and emphasis on developing and evaluating system designs in the context of the complete integrated plant design;
(2) Targeted technology research and development and system design studies driven by the requirements of the DEMO design concept and which respond to critical design feasibility and integration risks;
(3) Where possible, modest extrapolations from the ITER technology and physics basis to minimize development risks;
(4) Evaluation of multiple design options and parallel investigations for systems and technologies that have a high technical risk or novelty.

Lessons learnt from ITER are incorporated. The work is strongly focused on the design integration of a pulsed DEMO device that is largely extrapolated from ITER (single-null divertor, conventional H-mode).

Heat exhaust, materials and tritium self-sufficiency are covered by missions 2, 3 and 4. A fourth significant issue for DEMO is Remote Maintenance. A distinction here compared to ITER is to develop the idea to handle complete blanket sectors via a top port to reduce the time needed for any blanket replacement (in ITER individual blanket modules are handled via the equatorial port). Remote Maintenance schemes must be incorporated in the overall design from the very start as they affect the plant design and layout. The present DEMO baseline design [27] features only 16 toroidal field coils, to have enough space for the remote handling of the blanket sectors. Although most work is done on the DEMO baseline design, alternative designs are also being studied, based on different divertor concepts, featuring high-T_c_ superconducting coils and also a flexi-DEMO that can start in short pulse mode (approx. 1 h) and then later be upgraded to steady-state operation [28]. In this early phase of design, DEMO values of the magnetic fields and underlying magnets technologies are similar to those used in ITER. Higher field windings generate higher forces in the mechanical structures in and around the plasma and toroidal field coils, including the toroidal field coils themselves, and should be carefully assessed.

## 9. Cost of electricity

For fusion electricity to compete on the market it is important to keep the cost of electricity as low as possible.

The cost is largely driven by three factors: (1) the costs of the fuel, (2) the operational costs and (3) the cost of the infrastructure. A fusion reactor needs only a very limited amount of fuel at negligible costs. However, the operational costs (which are related to the availability of the plant) and the infrastructure are costly and therefore much attention is devoted to find ways to lower these costs. In the previous section it was already argued that the Remote Maintenance scheme should be optimized such that the time needed for replacements or repair are minimized, such that the plant has a higher availability. Novel developments that certainly will have a positive effect on the costs are additive manufacturing of complex components and virtual engineering in which virtual components are tested in a computer.

High-temperature superconductors can be operated at a somewhat higher temperature (20 K instead of 4 K) to reduce the cost of cryogenic cooling. Increasing the magnetic field has the drawback that the stainless steel support structure, which is needed to keep the coils in shape and to compensate the out-of-plane forces, will become more bulky. A simulation for the DEMO coils has led to the conclusion that an increase in the magnetic field strength from 12 T to 18 T would necessitate an increase of the radial depth of the stainless steel support structure from about 1 m to about 2.5 m. This needs to be added to the 1.7 m radial built at the high-field side, which was quoted in the Section on tritium breeding.

A recent optimization study (including safety, tritium breeding, remote maintenance, etc.) based on parameter variations in a systems code has come to the conclusion that a cost optimum is reached for a reactor with a major radius close to 9 m [29]. The maximum field on the central solenoid in these studies was limited to 13 T.

## 10. Stellarator

ITER and DEMO are based on the tokamak concept, which is by nature a pulsed device. The high current in the tokamak makes it prone to current-driven instabilities. Many of them can be stabilized or mitigated by adequate control techniques, although much work still needs to be done to completely avoid disruptions.

Europe is working on the stellarator as a long-term back-up strategy. A stellarator has all magnetic fields generated by external coils and is therefore by definition a continuous device. A stellarator has the second advantage that it doesn't feature the instabilities and disruptions that plague a tokamak plasma. But a stellarator is technically more complex than a tokamak and therefore the research (in terms of performance) is some decades behind the tokamak. Given its potential promise Europe has focused mission 8 on the stellarator; more specifically on Wendelstein 7-X (W7-X), the world's largest stellarator [30]. W7-X came into operation at the end of 2015, and the first experimental campaigns exceeded the expectations. In 2019 and 2020 it will be equipped with an actively cooled divertor such that long-pulse operation is possible.

## 11. Conclusion

The European Fusion Programme is coherent and addresses in an integrated way all elements that are needed to develop a fusion reactor. The Fusion Roadmap presents a realistic resource-loaded path towards the fusion power plant. Its time scale is strongly connected to the availability of resources. To continue the progress, the DEMO design needs to migrate from the present pre-conceptual design to a conceptual design by 2020. This will happen after a thorough Gate Review around 2020 in which a number of less promising design options will be deselected. On the critical path for DEMO—and in fact for any fusion reactor—is the construction of a 14 MeV neutron source. Ideally, the start of the DONES construction should take place early in the 2020s. DEMO operation is expected to start in the 2050s.

## Data accessibility

This article has no additional data.
